# National patient safety initiatives: Moving beyond what is necessary

**DOI:** 10.1186/2045-4015-1-20

**Published:** 2012-05-23

**Authors:** Eyal Zimlichman, David W Bates

**Affiliations:** 1 Division of General Medicine, Brigham and Women's Hospital, Boston, Massachusetts, USA; 2 Department of Medicine, Harvard Medical School, Boston, Massachusetts, USA; 3 Department of Health Care Policy and Management, Harvard School of Public Health, Boston, Massachusetts, USA

## Abstract

Ilan and Donchin have compared Israel and Canada's experiences in setting a national patient safety agenda. We broaden this comparison to include the U.S. experience, and suggest that there are three additional key steps which will be important in any national patient safety agenda, and which Israel in particular should consider. These are 1) using health information technology (HIT) to directly improve patient safety, 2) dissemination and broad use of checklists, and 3) measuring patient safety over time at the national level. Especially because of its already substantial commitment to HIT and well-developed HIT sector, Israel has a major opportunity to move forward rapidly in this area and to achieve broad impact on the safety front.

This is a commentary on http://www.ijhpr.org/content/1/1/19/

## Commentary

Making meaningful progress in advancing patient safety has become a global priority in the last decade. Key milestones were the ambitious agenda set in the U.S. by the Institute of Medicine (IOM) to reduce the number of patients who were hurt or died as a result of medical errors and adverse events [1] and more recently, establishment of the World Health Organization (WHO) global patient safety initiative [2]. National initiatives and policy play a critical role for nations to meet these ambitious goals. In the U.S., in the aftermath of the IOM report, a series of initiatives were launched including setting up federal patient safety organizations that have set goals and guidelines, engaging accrediting organizations to include patient safety programs for providers, funding for new research on patient safety and liability reforms that allowed providers to report safety events.

In Ilan and Donchin's report published in this issue of *The Israel Journal of Health Policy Research *the authors focus on the experience of two countries in setting a national patient safety agenda - Canada and Israel [3]. In the last decade, Canada has followed the U.S. and put in place a plan with ambitious goals aimed at significantly advancing the safety of patients, Israel has yet to set a formal national agenda and has pushed patient safety forward mainly through local initiatives and centers of excellence.

Ilan and Donchin go on to suggest what steps should be taken at the national level in Israel to establish an organized patient safety agenda. As immediate steps they stress the importance of establishing an organizational infrastructure starting from the national level and continuing with the major health care provider organizations, promoting research on patient safety through allocation of appropriate support for this work and emphasizing education of patient safety as a core curriculum component for health care professionals.

These important steps are indeed necessary, but in our opinion not sufficient to make a meaningful impact on a national level, as other countries are now learning. Canada has indeed taken many of the necessary steps to ensure a national patient safety agenda and infrastructure, as Ilan and Donchin rightfully point out. Still, a recent report by the Canadian Institute for Health Information has found that Canada performs on the lowest quartile among 17 reporting countries on rates of accidental puncture or laceration, obstetrical trauma as well as of foreign bodies left in during surgical procedures [4]. Similarly, in the U.S. three studies recently published have shown that rates of injury due to medical error have remained unchanged in the last decade [5-7] as all three conclude that the U.S. has made inadequate progress in patient safety.

What else is needed then to drive meaningful improvement in patient safety on a national scale? Our experience, as well as the emerging evidence, point to three steps that need to be a part of any national patient safety agenda. First, harnessing health information technology (HIT) to promote patient safety is pivotal because it extends to all providers. This is especially important for domains with robust evidence such as implementation of medication safety systems--computerized physicians order entry and bar-coding systems in particular, though we expect benefit will be demonstrated for other areas soon. With the high adoption rate of electronic health systems [8,9] Israel specifically could make significant strides on this in a relatively short time. Secondly, dissemination and use of checklists throughout all hospitals as part of a national initiative has the potential to substantially reduce morbidity and mortality, with some of the best data relating to surgical checklists and central line infection [10,11]. Down the road, implementation of checklists for other processes of care promises to hold additional benefit. With checklists, information technology could also help as a tool. Third, we think it will be important to measure patient safety nationally over time. While measuring patient safety metrics on a national scale has been done for some time especially in the OECD, often safety is assessed with claims data or chart reviews post hoc, with poor sensitivity and specificity [12]. By tapping into the abundant electronic data that exists within EHRs to measure patient safety, and designing EHRs so that they are able to systematically track adverse events in real time, it should be possible to make meaningful strides in improving patient safety.

Much still needs to be done to push patient safety forward globally. Although some countries have had a substantial head start in creating national patient safety infrastructure and policy, most have yet to realize substantial improvement. Policy makers need to learn what has worked and what has not and better understand what is yet needed. Harnessing HIT to promote patient safety holds much promise and could present Israel with an opportunity to turn the tide.

## Competing interests

The authors declare that they have no competing interests.

## Authors' information

Dr. Eyal Zimlichman is an internal medicine physician and a researcher at Brigham and Women's Hospital and the Harvard Medical School affiliated Center for Patient Safety Research and Practice and a lead researcher at the Department of Clinical Affairs at Partners Healthcare in Boston, U.S.A. Previously, he worked in Sheba Medical Center as the assistant to the CEO. Dr. David Bates is the Chief Quality Officer and Chief of General Internal Medicine at Brigham and Women's Hospital, Professor of Medicine, Harvard Medical School, and Professor of Health Policy and Management at the Harvard School of Public Health, Boston, MA.
